# Metabolic acidosis exacerbates pyelonephritis in mice prone to vesicoureteral reflux

**DOI:** 10.14814/phy2.14525

**Published:** 2020-10-08

**Authors:** Jeffrey M. Purkerson, Janine L. Corley, George J. Schwartz

**Affiliations:** ^1^ Pediatric Nephrology University of Rochester Medical Center Rochester NY USA; ^2^ Strong Children’s Research Center University of Rochester Medical Center Rochester NY USA

**Keywords:** acid–base physiology, inflammation, kidney collecting duct, urinary tract infection, uropathogenic *E. coli*

## Abstract

Acute pyelonephritis is a common, serious bacterial infection in children. The prevalence of acute pyelonephritis is due at least in part to vesicoureteral reflux (VUR). Although an association between abnormalities in electrolyte and acid–base balance and pyelonephritis is common in young children, the impact of metabolic acidosis (MA) on progression of acute pyelonephritis is not fully understood. In this study, the effect of MA on pyelonephritis was studied in C3H mouse strains prone to VUR. MA induced by ammonium chloride supplementation in food specifically impaired clearance of urinary tract infection with uropathogenic *Escherichia. coli* (UPEC‐UTI) in innate immune competent C3H strains (HeOuJ, HeN), whereas kidney UPEC burden in Tlr‐4‐deficient HeJ mice was unaffected. Antibody‐mediated depletion of myeloid cells (monocytes, neutrophil) markedly increased UPEC burden in the bladder and kidney confirming the pivotal role of neutrophils and tissue‐resident macrophages in clearance of UPEC‐UTI. MA concurrent with UPEC‐UTI markedly increased expression of cytokine (TNFα, IL‐1β, IL‐6) and chemokine (CXCL 1, 2, and 5) mRNA in isolated kidney CD cells and kidney neutrophil infiltrates were increased four‐ to fivefold compared to normal, UPEC‐infected mice. Thus, MA intensified pyelonephritis and increased the risk of kidney injury by impairing clearance of UPEC‐UTI and potentiating renal inflammation characterized by an elevated kidney neutrophil infiltrate.

## Graphical abstract

The burden of E coli was increased 3–4 order of magnitude higher in bladder and kidney in TL4‐sufficient mice made acidotic before infection.

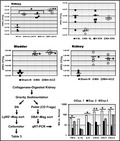

## 1 INTRODUCTION

Acute pyelonephritis is a common, serious bacterial infection in children with incidence in girls and boys under 8 years of ~8% and 2%, respectively (Montini, Tullus, & Hewitt, 2011). The prevalence of acute pyelonephritis is due at least in part to vesicoureteral reflux (VUR) or retrograde flow of urine from bladder to kidney. VUR facilitates ascension of bacteria to the kidney. The resulting infection triggers neutrophil influx and interstitial inflammation, which if unresolved, can lead to reflux nephropathy with chronic interstitial inflammation causing kidney fibrosis and chronic kidney disease (CKD). Young age, particularly infancy, has been deemed a risk factor for fibrotic scarring of the kidney (Peters et al., 2010; Representatives, 1999). Reflux nephropathy accounts for 12%–21% of all children with chronic renal failure (Chantler et al., 1980; Deleau, Andre, Briancon, & Musse, 1994; Furth et al., 2011).

Mouse models of VUR include C3H strains which exhibit up to 100% reflux (Bowen, Watt, Murawski, Gupta, & Abraham, 2013). Caudal ureteric bud formation along the mesonephric duct in C3H mice manifests as defects in the ureterovesical junction (UVJ) that in conjunction with shortened intravesical ureters facilitate retrograde flow of urine (Bowen et al., 2013; Fillion, Watt, & Gupta, 2014; Murawski et al., 2010). However, in C3H mice, VUR alone does not induce RN; urinary tract infection (UTI) comorbidity is well established (Bowen et al., 2013). Among the C3H congenic strains, HeN and HeOuJ are Tlr‐4 sufficient and prone to reflux (Hopkins, Gendron‐Fitzpatrick, Balish, & Uehling, 1998; Hopkins, Gendron‐Fitzpatrick, McCarthy, Haine, & Uehling, 1996; Li et al., 2017). In contrast, the C3H‐HeJ strain is innate immune compromised due to spontaneous mutation in the *tlr*4 gene, and thus HeJ mice are unable to clear infections of the urinary tract with gram‐negative bacteria (Haraoka et al., 1999; Ragnarsdottir & Svanborg, 2012; Shahin, Engberg, Hagberg, & Svanborg, 1987).

An association between abnormalities in electrolyte and acid–base balance and acute pyelonephritis is common in children under 3 years of age (Bertini et al., 2016); however, the impact of metabolic acidosis on progression of acute pyelonephritis is not fully understood. Ammonium chloride supplementation in water has been extensively used in animal models to study the pathophysiology of metabolic acidosis (McKinney & Burg, 1977; Nowik, Kampik, Mihailova, Eladari, & Wagner, 2010). In a seminal study of the impact of metabolic acidosis on urinary tract infection, ammonium chloride administration in water increased susceptibility to pyelonephritis in rodents; disposition toward infection was corrected by co‐administration of water, thus leading the authors to conclude that medullary hyperosmolality exacerbates acute pyelonephritis (Andriole, 1970). In the kidney collecting duct (CD), NH_4_Cl loading in water also elicits a vasopressin response and V2R‐mediated upregulation of AQP2 (Amlal, Sheriff, & Soleimani, 2004; Nowik et al., 2010). Thus, the influence of dehydration on susceptibility to pyelonephritis is mediated, at least in part, by vasopressin signaling via V2R that impairs uropathogenic *Escherichia coli* (UPEC)‐UTI clearance by attenuating Tlr‐4‐dependent innate immune responses to UPEC‐UTI (Chassin et al., 2007).

Recent studies in our laboratory has shown that acidosis in vivo and in vitro enhances innate immune defense via HIF‐1α‐dependent induction of antimicrobial peptide expression suggesting acidosis may confer increased resistance to UPEC infection (Peng, Purkerson, Freeman, & Schwaderer, 2020; Peng, Purkerson, Schwaderer, & Schwartz, 2017). In the study presented herein, we sought to clarify the effect of metabolic acidosis on the progression of acute pyelonephritis in C3H strains prone to VUR by administering ammonium chloride via supplementation of food, which induces metabolic acidosis in rodents without eliciting an AVP‐mediated dehydration response (Nowik et al., 2010). Herein, we report that in contrast to our recent in vitro study (Peng et al., 2020), metabolic acidosis concurrent with UPEC‐UTI markedly increased UPEC burden and exacerbated pyelonephritis specifically in innate immune competent C3H strains.

## 2 METHODS

### 2.1 Mice

C3H‐HeOuJ and HeJ (Jackson Laboratory, Bar Harbor, ME) or C3H‐HeNCrl mice (Charles River, Wilmington, MA) were purchased at 4–5 weeks and used for experimentation at 5–7 weeks of age. Mice were maintained on standard rodent chow (LabDiet 5010). Metabolic acidosis was induced via provision of special diet (Labdiet 5002 supplemented with 2% ammonium chloride) ad libitum for up to 7 days. Ammonium chloride supplemented with rodent chow was formulated and manufactured by Purina Mills Testdiet and distributed by Scott Pharma Solutions. In some experiments, 50 mg kg^−1^ day^−1^ acetozolamide was administered via subcutaneous insertion of Alzet® 1007D osmotic pumps (Durect Corp.) in conjunction with the 2% NH_4_Cl‐supplemented diet. All protocols and procedures involving mice were submitted to and approved by the University Committee on Animal Rights of the University of Rochester Medical Center (UCAR‐2016–023).

### 2.2 Acid–base status

Blood was collected from mice by tapping the retro‐orbital sinus under light anesthesia with a heparinized capillary pipette from which blood pH and s[HCO_3_^‐^] were measured utilizing iSTAT® G3+ Cartridges (Abbott Labs). Urine volume and pH were determined by collecting dark cycle urine under mineral oil from groups of two to four mice housed overnight in a metabolic cage with free access to food (powdered) and water. Urine pH was measured with pH strips and confirmed using a calomel pH electrode.

### 2.3 UPEC‐UTI

UPEC strain CFT073 was streaked onto tryptone phosphate agar plates and used to seed static UPEC cultures in tryptone phosphate. UPEC static culture was passaged and the secondary static culture was washed in HBSS and UPEC colony forming units (cfu) was determined by measuring OD600 nm (1_OD_ = 5 × 10^8^ cfu). Mice were deprived of water 1 hr before and after intrabladder inoculation of 0.5–1 × 10^7^ cfu in 50 µl of UPEC strain CFT073, an acute pyelonephritis isolate of *E. coli* (ATCC® 700928™) via the transurethral approach as described (Hung, Dodson, & Hultgren, 2009). UPEC burden (UPEC cfu/g tissue) was determined by culture of serial dilutions of tissue homogenates for which UPEC clearance is defined as <50 CFU/g tissue (Hains et al., 2014). Since bacterial burden is not normally distributed, statistical significance between groups was calculated with the Mann–Whitney *U* test (significance *p* < .05). When three comparisons were performed, statistical significance was established as *p* ≤ .02 using the Bonferroni correction of the 95% confidence interval.

### 2.4 Neutrophil/monocyte depletion

Purified, in vivo grade (reconstituted in PBS 7.0 containing no preservatives or stabilizers) rat IgGb isotype control antibody or monoclonal anti‐Ly6G/C (100 µg/100 µl, NIMP‐R14, BioXcell, Lebanon, NH) was injected intraperitoneally with UPEC CFT073 with respect to transurethral inoculation on days −1, 0, and +1. Ly6G/C isoforms are expressed on myeloid cells that include neutrophils and monocytes/macrophages (Privratsky et al., 2018; Rose, Misharin, & Perlman, 2012; Yu et al., 2016).

### 2.5 Kidney CD enrichment

Whole kidneys were minced in HBSS Ca/Mg free (Thermo Fischer Scientific), prior to physical dissociation utilizing two successive Multi_E_01 preset programs on a gentleMACS Dissociator (Miltenyi Biotec). Kidney homogenates were pelleted by centrifugation (300*g* for 10 min) and resuspended (5 ml/kidney) in collagenase digest solution {1 mg/ml collagenase IV (Thermo Fischer), 1 mg/ml soy trypsin inhibitor (Thermo Fischer), 0.25 mg/ml DNAse 1, type IV (Millipore Sigma) in HBSS buffer containing calcium chloride (1.3 mM),magnesium chloride (0.5 mM), and magnesium sulfate (0.4 mM), and incubated for 30 min at 37°C with gentle agitation. Following collagenase digestion, kidney tissue was placed on ice and physically dissociated utilizing a gentleMACS dissociator preset Multi_E_02 according to the manufacturer's recommended protocol. Kidney homogenates were passed through a 100‐µm sieve (MACS SmartStrainer, Miltenyi Biotec) and washed via centrifugation (300*g* for 10 min) prior to RBC lysis with ACK solution (ThermoFischer Scientific) according the manufacturer's recommended protocol. Following a second wash via centrifugation (300*g*), kidney cells/tubule fragments were resuspended in 10 ml of Ca/Mg‐free HBSS supplemented with 0.1% BSA and renal tubule fragments were enriched via gravity sedimentation for 15–20 min. Supernatants from gravity sedimentation were collected for magnetic sorting of Ly6G^+^ neutrophils as described below. The gravity sedimentation pellet was resuspended in PBS supplemented with Ca/Mg and 0.1% BSA (PBS/Ca/Mg, 10 mls) and CD fragments were enriched by first labeling with a 1:2,500 dilution of biotinylated Dolichos biflorus agglutinin (DBA‐biotin, Vector Labs, Burlingame, CA) for 30 min at 4°C with gentle rotation. DBA selectively binds to principal cells in the mouse CD and thus facilitates enrichment of CD fragments from mouse kidney (Holthöfer, 1988). Renal tubule fragments were pelleted by centrifugation (100*g* for 10 min) and resuspended in 1 ml 0.1% BSA PBS/Ca/Mg to which prewashed Dynabeads® Biotin Binder (62.5 µl suspension/kidney, ThermoFischer) were added and incubated for 30 min at 4°C with gentle rotation. CD fragments were magnetically sorted utilizing a DynaMag™ 2‐magnet (ThermoFischer). CD bound beads were washed three times to remove nonspecifically bound tubule fragments followed by a final wash in PBS without BSA. Total RNA was isolated from CD fragments utilizing the RNeasy Plus Mini Kit (Qiagen, Germantown, MD). CD enrichment was determined by quantification of relative abundance of AQP2 mRNA in DBA^(−)^ versus DBA^(+)^ fractions by qRT‐PCR as described below. Enrichment of AQP2 in DBA^(+)^ fractions was 106 ± 19 fold (*N* = 7) versus DBA^(−)^ renal tubule fractions. Relative abundance of SDF‐1 in CD isolated via lectin‐mediated magnetic from acidotic kidneys was 4.3 ± 0.4 fold (*N* = 4) compared to normal, which is consistent with previous studies analyzing expression in kidney tissue (Schwartz et al., 2015). Thus, CD preparations isolated via magnetic sorting with DBA lectin are suitable for analysis of CD gene expression.

### 2.6 CD Gene expression

First‐strand cDNA synthesis utilizing 100–500 ng of total RNA was carried out using the Superscript™ III First Strand Synthesis System (ThermoFischer). Relative abundance of mRNA’s (GAPDH, AQP2, E‐Cadherin, chemokines, cytokines) was determined by qRT‐PCR utilizing TaqMan™ gene expression master mix (ThermoFischer), TaqMan™ primer, probes specific for mouse homologues and an ABI 7500 instrument (Applied Biosystems). PrimeTime® Std qPCR assays (FAM‐TAMRA) for *Mus musculus* glyceraldehyde‐3‐phosphate dehydrogenase (Gapdh), NCBI Reference Sequence: NM_008084.3, *Mus musculus* aquaporin 2 (Aqp2), mRNA, NCBI reference Sequence: NM_009699.3, and *Mus musculus* cadherin 1 (Cdh1), mRNA NCBI Reference Sequence: NM_009864.3 were designed with PrimerQuest® and the respective primer set 1 was synthesized by Integrated DNA Technologies, Inc.). Quantitation of cytokine/chemokine mRNA abundance was accomplished utilizing commercially available TaqMan® Real Time PCR assays (FAM‐MGB probes, ThermoFischer Scientific) listed in Table 1. Relative abundance of mRNA’s in DBA^(−)^ versus DBA^(+)^ CD or DBA^(+)^ CD isolated from normal versus acidotic kidneys was determined by the ΔΔCt method utilizing Gapdh or Cdh1 as a reference. Statistical significance of fold change in chemokine/cytokine mRNA abundance in DBA(+) CD cells isolated from normal versus acidotic kidneys was determined by Mann–Whitney *U* test (significance at *p* < .05).

**TABLE 1:** Cytokine/Chemokine TaqMan® Real Time PCR assays (ThermoFischer)

IL‐1β	TNFα	IL‐6	CXCL 1	CXCL 2	CXCL 5	CXCL 12
Mm00434228_m1	Mm00443258_m1	Mm00446190_m1	Mm04207460_m1	Mm00436450_m1	Mm00436451_g1	Mm00445553.m1

### 2.7 Enumeration of Neutrophil Infiltrates

In preparation for isolation of Ly6G^+^ neutrophils from collagenase‐digested kidney, gravity sedimentation supernatants from two mice were pooled and pelleted via centrifugation (300*g* for 10 min) and resuspended in 1–2 ml of isolation buffer (HBSS, Ca/Mg free, supplemented with 1% FCS). In order to prevent antibody binding via Fc receptors, magnetic sorting was performed via the direct technique in which Dynabeads™ Sheep anti‐Rat IgG (DynabeadsR, ThermoFischer) were precoated with monoclonal anti‐Ly6G^+^ (1A8, BioXcell) prior to incubation with gravity sedimentation supernatants. Briefly, 1A8 antibody (2 µg) was incubated with 50 µl of prewashed DynabeadsR for ≥30 min at 4°C with gentle agitation, after which beads were washed 3X via magnetic separation to remove excess antibody. Positive isolation of Ly6G^+^ cells was accomplished by adding 1A8‐coated DynabeadR to the kidney cell suspension and incubating for 20–30 min at 4°C with gentle agitation via rotation. Prior to magnetic separation 1 ml of isolation buffer was added to limit trapping of unbound cells and magnetic bead‐bound cells were washed 3X to remove nonspecifically bound cells. Live/Dead Ly6G^+^ bead‐bound cells were enumerated by diluting a 25 µl aliquot 1:1 in ViaStain™ AO/PI Viability Stain and fluorescent imaging utilizing a Cellometer K2 Image Cytometer (Nexcelom Bioscience).

## 3 RESULTS

### 3.1 Metabolic acidosis impairs clearance of UPEC‐UTI in mice prone to vesicoureteral reflux

**FIGURE 1:**
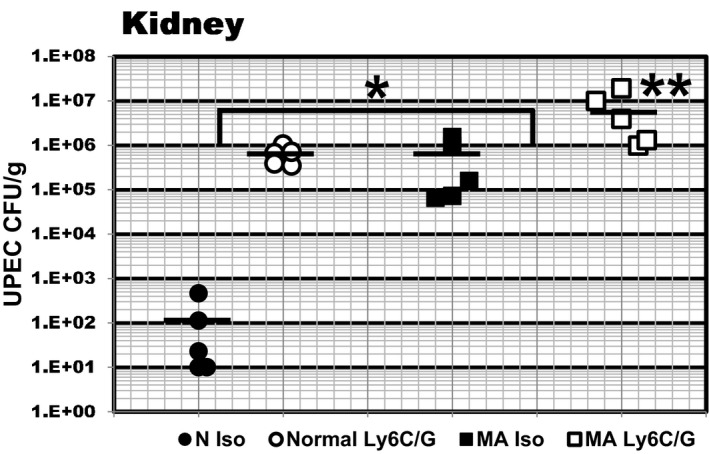
Metabolic acidosis (MA) impairs clearance of UPEC‐UTI in mice prone to vesicoureteral reflux (VUR). C3H HeN (5/group) were fed normal rodent chow or rodent chow supplemented with 2% NH_4_Cl (MA), from day −2 to +3 with respect to bladder instillation of with 5 × 10^6^ cfu/50 µl cfu UPEC strain CFT073 on day 0. For antibody‐mediated depletion of Ly6G/C+ cells, C3H‐HeN mice (5/group) were administered rat IgG2b isotype control 100 µg i.p. (Iso) and/or NIMP‐R14, monoclonal anti‐Ly6G/C 100 µg I.P. aon days −1, 0 and +1.Kidneys were harvested 3 days post‐infection (dpi) and UPEC burden determined by plating serial dilutions of tissue homogenates. Lines delineate average bacterial burden. *p < .02 versus Normal, **p < .05 versus MA isotype control; Mann–Whitney *U*‐TEST. Similar results were observed in bladder homogenates; data not shown.

In the experiment shown in Figure 1, UPEC burden in mice fed NH_4_Cl supplemented food was compared to mice in which neutrophil/monocytes populations had been depleted. Ammonium chloride supplementation in food‐induced metabolic acidosis (2% NH_4_Cl: s[HCO_3_^−^] = 17 ± 0.6 mM mean ± SE; *p* < .05 versus normal rodent diet: s[HCO_3_^−^] = 22.2 ± 0.7 mM, Table 2). Innate immune competent HeN mice (5/group) were either fed normal rodent chow or NH_4_Cl (2%) supplemented diet ad libitum from day −2 and administered isotype control (rat IgG2b) or monoclonal anti‐Ly6G/C (NIMP‐14) 100 µg/day i.p.. on days −1, 0, and +1 with respect to transurethral inoculation with UPEC (5 × 10^6^ cfu) on day 0, and UPEC burden was assessed 3 days post infection (dpi). It is important to note that this antibody‐mediated depletion protocol results in an incomplete reduction of myeloid populations as repeated antibody dosage of 300–500 µg antibody is required for complete elimination of myeloid populations largely due to rapid restoration of neutrophils via hematopoiesis in bone marrow (Pollenus et al., 2019). However, administration of as little as 10 μg monoclonal antibody has been shown to reduce bladder neutrophil infiltrates (Hannan et al., 2014). UPEC Burden (mean ± SE) in either acidotic (5.6E5 ± 6.6E5) or neutrophil depleted mice (6.3E5 ± 1.2E5) was three orders of magnitude higher than normal mice administered isotype control (1.2E2 ± 8.7E1; see Figure 1). Similar results were observed in bladder and NH_4_Cl‐loading of another innate immune competent strain, C3H‐HeOuJ mice (data not shown). Metabolic acidosis in neutrophil/monocyte depleted mice increased UPEC burden by another order of magnitude (7.0E6 ± 7.6E6; *p* < .05 versus MA alone; Mann–Whitney *U* test; see Figure 1). These results demonstrate that MA markedly increases UPEC burden in innate immune competent C3H mice, and that this effect was comparable to partial depletion of myeloid cells that constrain bacterial infections (Haraoka et al., 1999). The combination of MA plus neutrophil/monocyte depletion had a smaller additional effect on UPEC burden.

**TABLE 2:** Acid/base Parameters

Condition	Blood pH	s[HCO_3_]^‐^	N (iSTAT)	Urine pH	N Ur pH
Normal	7.2 ± 0.01	22.2 ± 0.7	23	6.8 ± 0.1	7
MA	7.1 ± 0.02*	17.0 ± 0.6^†^	9	5.8 ± 0.2*	4
MA + ACZ	7.0 ± 0.01*	14.4 ± 0.42*	3	6.75 ± 0.3	3

Note

MA, Metabolic acidosis; ACZ, acetazolamide 50 mg kg^‐1^ day^‐1^ via Alzet® Osmotic pump mean ± SE.

* *p* < .05 versus normal.

^†^ *p* < .001, t‐test.

### 3.2 Metabolic acidosis does not increase kidney UPEC burden in Tlr‐4‐deficient mice

**FIGURE 2:**
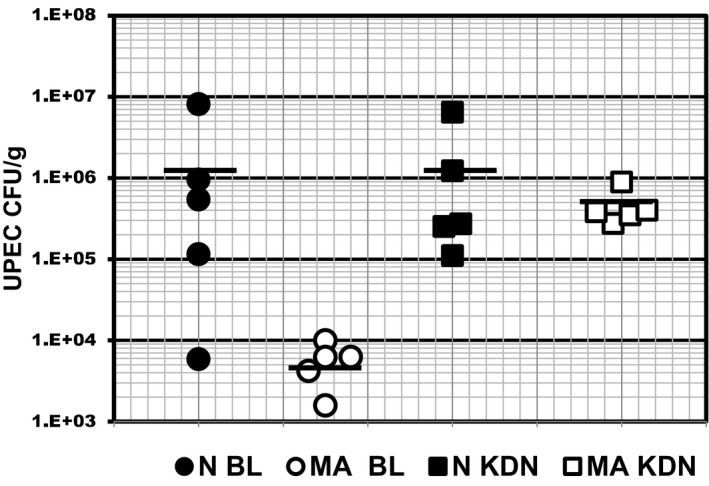
Metabolic acidosis (MA)does not increase kidney UPEC burden in Tlr‐4‐deficient mice. Tlr‐4‐deficient HeJ mice (5/group) were fed normal rodent chow (normal) or rodent chow supplemented with 2% NH_4_Cl (MA) from day −2 to +3 with respect to bladder instillation of with UPEC strain CFT073 5 × 10^6^ cfu/50 μl on Day 0. Bladder and kidneys were harvested 3 dpi and UPEC burden determined by plating serial dilutions of tissue homogenates. Lines delineate average bacterial burden

**FIGURE 3:**
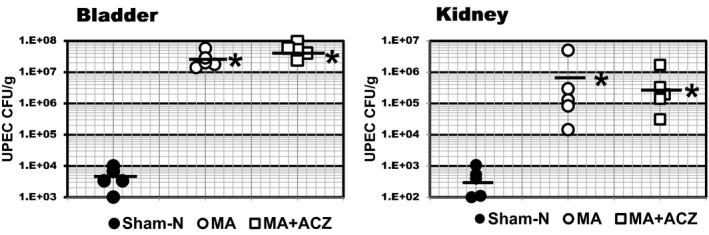
Urine acidification does not affect UPEC burden. C3H‐HeN mice (5/group) were fed normal rodent chow (5010, normal), rodent chow 5002 supplemented with 2% NH_4_Cl (Acidosis), or 2% NH_4_Cl‐supplemented diet in conjunction with 50 mg kg^−1^ day^−1^ acetozolamide administered via Alzet® osmotic pump from day −2 to +3 with respect to bladder instillation of with 10^7^ cfu/50 ul UPEC strain CFT073 on Day 0. Kidneys were harvested day +3 PI and UPEC burden determined by plating serial dilutions of tissue homogenates. Lines delineate average bacterial burden. *p < .02 versus Normal; Mann–Whitney *U*‐TEST. Sham‐*N* = sham surgery normal diet, MA = Metabolic Acidosis, MA + ACZ = metabolic acidosis in conjunction with 50 mg kg^−1^ day^−1^ acetazolamide

One explanation for the additional effect of MA on UPEC burden in mice partially depleted of myeloid cells is attenuation of residual neutrophil/monocyte function. The next set of experiments was designed to determine whether MA increases UPEC burden in C3H‐HeJ mice that are Tl4 deficient due to a spontaneous mutation in the *tlr*4 gene (Murawski et al., 2010). Tlr‐4‐deficient HeJ mice are unable to clear UPEC UTI due in large part to diminished neutrophil recruitment to bladder and kidney (Chassin et al., 2006; Haraoka et al., 1999; Shahin et al., 1987). C3H‐HeJ mice (5/group) were administered NH_4_Cl (2%) supplemented diet ad libitum from day −2 through 3 dpi with respect to transurethral inoculation with 5X10^6^ cfu (HeJ) UPEC (CFT073) on day 0 and UPEC burden in bladder and kidney was determined 3 dpi. Consistent with the pivotal role of Tlr‐4 in innate immune defense against gram‐negative bacteria (Janeway & Medzhitov, 2002), kidney UPEC burden in HeJ mice was higher than in HeN mice fed a normal diet (UPEC Burden [cfu/g]: Normal HeJ kidney = 2E6 ± 1E6 compare Figures 1, 2, 3). However, metabolic acidosis did not further increase bladder or kidney UPEC burden in Tlr‐4‐deficient HeJ mice (Figure 2). Although it did not reach statistical significance, an apparent trend toward decreased UPEC burden in bladder of acidotic HeJ mice likely resulted from increased diuresis caused by ammonium chloride supplementation of the rodent diet (dark cycle urine volume [ml/mouse per day]: 2% NH_4_Cl diet = 1.5 ± 0.2,; *p* < .05 versus normal rodent diet = 0.80, Table 2). Schwaderer and colleagues came to a similar conclusion regarding the effect of diuresis caused by intraperitoneal administration of acetazolamide or furosemide (Ketz et al., 2020). Relative abundance of AQP2 mRNA levels in DBA + CD preparations isolated from acidotic mice were 0.5 ± 0.1 times (*n* = 4) the AQP2 expression level in mice fed the standard rodent diet demonstrating that, consistent with results reported by Nowik et al. (2010), NH_4_Cl supplementation did not upregulate AQP2 expression in the kidney collecting duct. Thus, the marked enhancement of pyelonephritis by metabolic acidosis induced by NH_4_Cl‐loading cannot be directly attributed to attenuation of Tlr‐4‐dependent responses by signaling via AVP‐V2R ( Chassin et al., 2007). Specific impairment of UPEC‐UTI clearance in innate immune competent mice indicates that metabolic acidosis attenuates some aspect of Tlr‐4‐dependent innate immune defense that includes neutrophil recruitment and/or function (Chassin et al., 2006; Haraoka et al., 1999; Shahin et al., 1987).

### 3.3 UPEC Burden is not influenced by urine acidification

The observation that MA did not influence kidney UPEC burden in Tlr‐4‐deficient HeJ mice (See Figure 2) suggests that urine acidification per se (2% NH_4_Cl: urine pH 5.8 ± 0.2, *p* < .05 versus normal diet pH 6.8 ± 0.1, Table 2) is not a major contributor to UPEC clearance from kidney. Consistent with this supposition, neutralization of urine pH in the setting of metabolic acidosis (See Table 2) via concurrent administration of 50 mg kg^−1^ day^−1^ acetazolamide via Alzet® osmotic pumps with ammonium‐chloride supplementation did not affect UPEC burden in HeN mice compared to NH_4_Cl administration alone (Figure 3). UPEC burden in acidotic mice was three to four orders magnitude higher in bladder and kidney compared to normal control irrespective of concurrent treatment with acetazolamide.

### 3.4 CD chemokine/cytokine production is increased by metabolic acidosis concurrent with UPEC‐UTI

**FIGURE 4:**
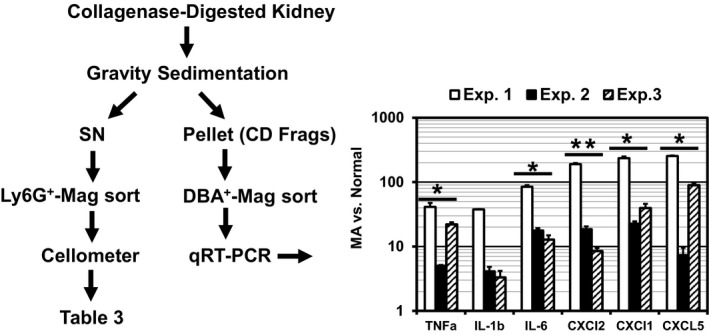
Metabolic acidosis (MA) concurrent with UPEC‐UTI markedly increases CD inflammation. C3H‐HeN or HeOuJ were fed normal rodent chow (normal) or rodent chow supplemented with 2% NH_4_Cl (MA), from day −2, or −1 to +3 with respect to bladder instillation of with 1 × 10^7^ cfu/50 μl UPEC strain CFT073 on day 0. Kidneys were harvested 3 dpi and kidney CDs were isolated by DBA‐lectin‐mediated magnetic sorting of collagenase‐digested kidney. Relative cytokine/chemokine abundance in RNA isolated from DBA^+^ CDs was determined by qRT‐PCR and ΔΔCt was calculated utilizing GAPDH as a reference gene. Each bar represents ratio of MA to normal control (mean ± *SD*, 2 mice/condition); *p < .01, **p < .05 versus normal; Mann–Whitney *U*‐Test

The next experimental series was designed to determine the extent to which metabolic acidosis concurrent with UPEC‐UTI exacerbates renal inflammation. Tlr‐4 signaling in response to its ligand, lipopolysaccharide (LPS), a cell component of gram‐negative bacteria, elicits cytokine and chemokine responses as key facets of the tissue response to infection and injury (Chassin et al., 2006; Janeway & Medzhitov, 2002; Zeytun, Chaudhary, Pardington, Cary, & Gupta, 2010). Since the renal CD is the first nephron segment to encounter an ascending UPEC‐UTI, the relative abundances of cytokines (TNFα, IL‐1β, IL‐6) and chemokines (CXCL 1, 2, and 5) mRNA were quantitated in RNA isolated from DBA^+^ CDs enriched from collagenase‐digested kidney via lectin‐mediated magnetic sorting as described in Methods and outlined in Figure 4. Cytokine and chemokine mRNA abundance was as much as one to two orders of magnitude higher in CDs isolated from acidotic UPEC‐infected mice compared to normal infected mice. The variation in chemokine/cytokine mRNA levels in acidotic versus normal CDs from UPEC‐infected mice (e.g., IL‐1β and CXCL 2) was largely due to the extent of inflammation in situ or lack thereof in normal infected mice. These results demonstrate that increased UPEC burden due to concurrent metabolic acidosis markedly increases CD inflammatory stress in mice prone to vesicoureteral reflux.

### 3.5 Metabolic acidosis increases the kidney neutrophil infiltrate in response to UPEC‐UTI

Chemokines (CXCL 1, 2, and 5) induced by UPEC‐UTI concurrent with metabolic acidosis (Figure 4) are CXCR 2 ligands that play a prominent role in recruitment of neutrophils to sites of infection (Rajarathnam, Schnoor, Richardson, & Rajagopal, 2019; Svensson, Irjala, Svanborg, & Godaly, 2008). Therefore, whether enhanced chemokine production in CDs was accompanied by increased kidney neutrophil infiltrate was examined in parallel studies by enumerating neutrophils isolated from collagenase digested kidney CD cells via magnetic sorting with monoclonal anti‐Ly6G antibody (1A8) as outlined in Figure 4. In three independent experiments, Ly6G^+^ kidney neutrophil infiltrates isolated from kidneys of normal infected mice were comprised of (mean ± SE) 7E5 ± 2E5 cells with 59.1 ± 5.8% viability (See Table 3). Neutrophil Infiltrates in acidotic mice were comprised of 3E6 ± 1E6 Ly6G+ cells or an increase of 4.5 ± 0.6 fold (*N* = 3; *p* < .01 versus normal, t‐test) consistent with higher levels of chemokine expression in CDs isolated from kidneys of acidotic infected mice versus normal infected mice (Figure 4). Whether the number of Ly6G^+^ neutrophil infiltrates in acidotic mice is commensurate with the chemokine responses elicited by the high UPEC burden observed in Figures 1, 3 and 4 is unclear. Interestingly, viability of Ly6G^+^ cells isolated from kidneys of acidotic mice was consistently higher at 71.8 ± 0.8% (*p* < .05 versus normal, t‐test). Reduced viability of neutrophil infiltrates in kidneys from normal mice may result from formation of neutrophil extracellular traps (NETosis). Suicidal NETosis is a key component of neutrophil bactericidal activity that involves release of a fibrous network composed of decondensed chromatin, serine proteases and antimicrobial peptides that functions to entrap and kill bacteria (Brinkmann et al., 2004; Castanheira & Kubes, 2019; Sollberger, Tilley, & Zychlinsky, 2018). Thus, increased viability of kidney neutrophil infiltrates may reflect attenuated neutrophil bactericidal function (e.g., NETosis) in acidotic mice.

**TABLE 3:** Kidney neutrophil infiltrate is increased by metabolic acidosis during UPEC‐UTI

	Normal				Metabolic acidosis				Fold
	Live	Dead	%Viable	Total	Live	Dead	%Viable	Total	Diff.
Exp. 1	4.8E5	4.6E5	51.0%	9.5E5	2.3E6	1.2E6	66.1%	3.5E6	3.7
Exp. 2	1.5E5	1.1E5	57.6%	2.7E5	7.1E5	3.9E5	64.4%	1.1E6	4.1
Exp. 3	6.6E5	2.7E5	70.6%	9.3E5	4.6E6	8.2E5	84.8%	5.4E6	5.8

Note

Neutrophils were isolated from a pool of supernatants from collagenase digested kidneys (4 kidneys from 2 mice/condition) by magnetic sorting utilizing monoclonal anti‐Ly6G (1A8) as outlined in Figure 4. Neutrophils (live/dead) were enumerated via acridine orange/propidium iodide (AO/PI) staining and analysis utilizing a Cellometer K2 Fluorescent Viability Cell Counter (Nexcelom Bioscience). Fold difference versus normal (Mean ± SE) = 4.5 ± 0.6 *p* < .01 versus normal, *T*‐test.

## 4 DISCUSSION

A major finding of this study is that metabolic acidosis induced by ammonium chloride supplementation of food markedly impairs clearance of UPEC‐UTI (Figure 1). UPEC burdens in innate immune competent C3H mice (HeN, HeOuJ) were orders of magnitude higher than in control mice infected similarly and fed a standard rodent diet (Figures 1 and 3). This result was unexpected based on previous studies from our laboratory and others describing contributions of α‐intercalated cells and acidosis to innate immune defense of the CD against UPEC infection. We previously reported that metabolic acidosis induced via ammonium chloride supplementation of drinking water increased expression of the antimicrobial peptide (AMP), cathelicidin, in the rabbit CD (Peng et al., 2017) and that acid‐loading of mouse CD cells in vitro induced AMP expression and enhanced resistance to UPEC infection via HIF‐1α (Peng et al., 2020). Consistent with these results, Lin et al. reported that pharmacological stabilization of HIF‐1α in urothelial cells in vitro and in vivo induced AMP expression and reduced UPEC burden (Lin et al., 2015) However, it is important to note that HIF‐1α stabilization by acidosis is modest compared to HIF‐1α expression induced by hypoxia or pharmacological inhibition of prolyl‐hydroxlase (Nadtochiy et al., 2016; Peng et al., 2020). Thus, acidosis elicited only marginal gains in innate immune defense; a two‐three fold induction of AMP expression and ~30% increase in resistance to infection (Peng et al., 2017, 2020).

The dramatic effect of metabolic acidosis on UPEC burden is also in apparent contradiction with previously reported contributions of α‐ICs and urine acidification in protection against pyelonephritis. Paragus et al. reported that α‐ICs of the CD play a pivotal role in innate immune defense of the kidney via production of lipocalin‐2 (NGAL) and urine acidification in response to UPEC‐UTI (Paragas et al., 2014). While the antimicrobial effect of NGAL is well established, the contribution of urine acidification to protection against UPEC‐UTI is dubious. Conventional wisdom posits that urine acidification limits ascension of UTI, since urine acidification to a pH ≤ 6 limits UPEC growth (Paragas et al., 2014; Shohl & Janney, 1917). However, acidic pH reduces bactericidal activity of antimicrobial peptides (Johansson, Gudmundsson, Rottenberg, Berndt, & Agerberth, 1998), and can compromise other aspects of epithelial barrier function (Berkebile & McCray, 2014; Torres, Demirdjian, Vargas, Goodale, & Berwin, 2017), indicating that aspects of innate immune defense are pH dependent. Ammonium chloride administration in food acidified urine by 1 pH unit (i.e., pH 6.8 to 5.8, see Table 2), under pathophysiological conditions that markedly increased UPEC burden (Figures 1 and 3). Furthermore, neutralization of urine pH in the setting of metabolic acidosis by co‐administration of acetazolamide (Table 2, Figure 3) did not alter UPEC burden compared to metabolic acidosis alone. Collectively, these results indicate the urine acidification is inconsequential for progression of pyelonephritis.

In a related study, Schwaderer and colleagues reported that carbonic anhydrase (CA) 2‐deficient mice exhibit metabolic acidosis associated with a slight deficit in urine acidification likely due to reduced numbers of ICs (Breton et al., 1995). Although AMP (Lcn2, Camp) expression in kidney was elevated, *Car2* ^−/−^ mice had significantly increased UPEC burden compared to wildtype mice similarly infected (Hains et al., 2014). Normalization of serum bicarbonate by base supplementation of drinking water failed to improve resistance to UPEC‐UTI in *Car2* ^−/−^ mice leading the authors to conclude that reduced numbers of ICs rather than systemic acidosis increased susceptibility to pyelonephritis. A major limitation of this study was wide ranging pathophysiology due to CA2‐deficiency in multiple organ systems. In a subsequent study, transplantation of *Car2* ^−/−^ kidneys into normal syngeneic hosts reportedly increased UPEC burden, excluding CA2‐deficiency in other organ systems including myeloid cells as an explanation for increased susceptibility to UPEC infection (Ketz et al., 2020). However, the impact of systemic acidosis due to kidney CA2 deficiency was not addressed in the latter study. Although metabolic acidosis increases α‐IC function and the apparent number of α‐ICs in the mouse cortical CD via SDF‐1 signaling through CXCR4 (Schwartz et al., 2015), results presented herein clearly demonstrate that metabolic acidosis markedly increases UPEC burden in mice prone to VUR. Therefore, other aspects of pathophysiology associated with metabolic acidosis supersede any benefit of AMP production by α‐ICs (Paragas et al., 2014; Saxena et al., 2018, 2019; Spencer, Schwaderer, Becknell, Watson, & Hains, 2014).

Another key finding of this study was that metabolic acidosis specifically increases UPEC burden in innate immune competent mice; in contrast, kidney UPEC burden in Tlr‐4‐deficient HeJ mice, albeit higher than Tlr‐4‐sufficient HeN mice, was unaffected by metabolic acidosis (See Figure 2). Although not statistically significant, there was an apparent trend toward reduced UPEC burden in bladders of acidotic HeJ mice that likely resulted from ammonium chloride induced diuresis (Table 2), which may have facilitated removal of UPEC from the urinary tract. More importantly, the selective effect on Tlr‐4‐sufficient C3H strains presented in Figure 2 indicates the metabolic acidosis compromises some aspect of Tlr‐4 dependent innate immune defense. Although recent studies from our laboratory and others have focused on the role of AMPs produced by renal epithelial cells in innate defense against UPEC‐UTI (Eichler et al., 2019; Paragas et al., 2014; Peng et al., 2017, 2020; Saxena et al., 2018, 2019), results in Figure 1 illustrates how depletion of Ly6G/C^+^ myeloid cells (e.g. monocytes, neutrophils) markedly increased UPEC burden in HeN mice. This result confirms the essential role of myeloid cells, including neutrophils, in UPEC‐UTI clearance. Production of cytokines and chemokines that recruit neutrophils to sites of infection and injury is a pivotal contribution of Tlr‐4 signaling to innate immune defense against bacterial pathogens (Janeway & Medzhitov, 2002; Medzhitov, 2001; Zeytun et al., 2010). High bacterial burden in Tlr‐4‐deficient HeJ mice is due to attenuated recruitment of neutrophils to the urinary tract in response to UTI (Chassin et al., 2006; Haraoka et al., 1999; Ragnarsdottir & Svanborg, 2012; Shahin et al., 1987). Selective inhibition of UPEC‐UTI clearance in Tlr‐4‐sufficient C3H strains is consistent with the hypothesis that metabolic acidosis attenuates a key aspect(s) of Tlr‐4‐dependent responses to UPEC‐UTI.

The impact of metabolic acidosis on neutrophil recruitment and/or function is not fully understood. The influence of media pH or acidemia on neutrophil function has been studied extensively in vitro with mixed results (Erra Diaz, Dantas, & Geffner, 2018). Metabolic acidosis induced by ammonium chloride‐loading is associated with modest changes in blood pH (See Table 2), casting doubt as to whether an influence of metabolic acidosis on neutrophil function is strictly a pH‐dependent phenomenon. Furthermore, in vitro models do not recapitulate systemic metabolic derangements or hormonal responses associated with metabolic acidosis. Specifically, ammonium chloride acidosis is associated with major alterations in glutamine interorgan exchange and derangement of glutamine metabolism during which the kidney becomes a major site of glutamine uptake and catabolism (Taylor & Curthoys, 2004). Metabolic stress of acidosis may deplete systemic glutamine reserves resulting in a reduction in Gln:Glu ratio which could impair neutrophil function in the renal medulla, the latter can become ischemic during an ascending UPEC‐UTI (Basiratnia, Noohi, Lotfi, & Alavi, 2006; Halevy et al., 2004; Stogianni et al., 2007). In ischemic tissues, neutrophils catabolize Gln as a carbon source for free radical and cytokine production via anaplerotic shunting of Gln to the Krebs cycle (Cruzat, Macedo Rogero, Noel Keane, Curi, & Newsholme, 2018; Metallo et al., 2011; Newsholme et al., 1999; Wang et al., 2019). In a recent study investigating the impact of a high‐salt diet on susceptibility pyelonephritis, Jobin et al. reported that glucocorticoid‐mediated suppression of neutrophil function exacerbates pyelonephritis in mice (Jobin et al., 2020). Elevated corticosterone levels in acidotic rodents has been reported (May, Kelly, & Mitch, 1986) and therefore stimulation of glucocorticoid production by metabolic acidosis may represent an alternative mechanism for increased susceptibility to pyelonephritis. Additional experiments beyond the scope of the present study are required to assess the impact of metabolic acidosis on neutrophil function.

Results in Figure 4 and Table 3 demonstrate chemokine and cytokine production in the CD, and kidney neutrophil infiltrates are elevated in acidotic, infected HeN mice consistent with elevated UPEC burden (Svensson et al., 2008). However, impaired clearance in these mice suggests that the neutrophil response and/or function is attenuated by metabolic acidosis. Consistent with this hypothesis neutrophil viability was higher in acidotic mice suggesting reduced formation of neutrophil extracellular traps via suicidal NETosis, a key component of neutrophil bactericidal activity. Perhaps more importantly, results presented in Figure 4 and Table 3 demonstrate that if left uncorrected metabolic acidosis increases the severity and duration of pyelonephritis and thus will likely worsen renal tubular injury caused by inflammation. Neutrophils are the most abundant immune cells in circulation and first responders to sites of infection and injury; they play a pivotal role in clearance via phagocytosis coupled to bactericidal activity (Bonavia & Singbartl, 2018). Neutrophil bactericidal mechanisms are not pathogen‐specific and thus have the potential to cause damage to host cells and tissues. In a mouse model (e.g. C3H‐HeOuJ) of chronic UPEC infection, renal fibrosis was linked to inflammation characterized by recruitment of phagocytes (e.g., neutrophils, macrophages) to sites of infection (Li et al., 2017). Dysregulated neutrophil function contributes to the pathogenesis of several immuno‐inflammatory disease states ranging from atherosclerosis to cancer (Leslie, 2020). Myeloperoxidase is the most abundant protein in azurophilic granules of phagocytes where it plays a pivotal role in phagocyte bactericidal activity via production of reactive oxygen species (ROS) (Segal, 2005). Increasing the magnitude and duration of the kidney neutrophil infiltrate and thus ROS production can overwhelm renal antioxidant systems resulting in oxidative stress and renal tubular injury (Okamura & Himmelfarb, 2009). Neutrophil elastase (NE) is another enzymatic component of neutrophil azurophilic granules that plays a pivotal role in NETosis, a component of neutrophil bactericidal activity (Kolaczkowska et al., 2015). Dysregulated NE activity associated with neutrophil infiltrates has been linked to pulmonary fibrosis (Sallenave, 2015) and CKD progression (Bronze‐da‐Rocha & Santos‐Silva, 2018). Furthermore, in addition to facilitating bacterial clearance, NETosis provides ligands and epitopes for positive feedback stimulation of both innate and adaptive immune responses which can lead to renal inflammatory disease (Gupta & Kaplan, 2016 **).** Thus, concurrent metabolic acidosis exacerbates pyelonephritis by both impeding clearance of UPEC‐UTI and enhancing renal inflammation characterized by an elevated kidney neutrophil infiltrate.

## CONFLICT OF INTEREST

GJS received consulting fees from Tricida and Astra Zeneca.

## AUTHOR CONTRIBUTIONS

JMP designed and executed experiments, and analyzed data in consultation with GJS. JMP prepared figures and wrote the manuscript which were reviewed and edited by GJS.
